# Calf circumference was negatively associated with all-cause mortality among the Chinese centenarians: a prospective study with a 5-year follow-up

**DOI:** 10.1007/s40520-024-02850-3

**Published:** 2024-10-05

**Authors:** Zhigao Sun, Hengbo Zhang, Xiaofei Liu, Xiaobing Wang, Qiong Liu, Yali Zhao, Yan Nie, Dongzong Huang, Shihui Fu

**Affiliations:** 1 Traditional Chinese Medicine Department, Hainan Hospital of Chinese People’s Liberation Army General Hospital, Sanya, China; 2 Department of Burn and Plastic Surgery, Hainan Hospital of Chinese People’s Liberation Army General Hospital, Sanya, China; 3 Department of Rheumatology and Immunology, Hainan Hospital of Chinese People’s Liberation Army General Hospital, Sanya, China; 4 Department of Military Education and Training, Naval Aeronautical and Astronautical University, Yantai, China; 5 Medical Care Center, Hainan Hospital of Chinese People’s Liberation Army General Hospital, Sanya, China; 6 Central Laboratory, Hainan Hospital of Chinese People’s Liberation Army General Hospital, Sanya, China; 7 Gastroenterology Department, Hainan Hospital of Chinese People’s Liberation Army General Hospital, Sanya, China; 8 Medical School of Chinese People’s Liberation Army, Beijing, China; 9 Department of Stomatology, The First Medical Centre of Chinese People’s Liberation Army General Hospital, Beijing, China; 10 Department of Cardiology, Hainan Geriatric Disease Clinical Medical Research Center, Hainan Branch of China Geriatric Disease Clinical Research Center, Hainan Hospital of Chinese People’s Liberation Army General Hospital, Sanya, China; 11 Department of Geriatric Cardiology, Chinese People’s Liberation Army General Hospital, Beijing, China

**Keywords:** All-cause mortality, Calf circumference, Chinese centenarians, Human longevity, Sarcopenia

## Abstract

**Background:**

Sarcopenia is an aging-related disorder characterized by a loss of muscle mass and function. Calf circumference (CC) is a useful surrogate marker of muscle mass and function. This prospective study was designed to investigate the association between CC and all-cause mortality during a follow-up for 5 years in the Chinese centenarians.

**Methods:**

The China Hainan Centenarian Cohort Study (CHCCS) is conducted in 18 cities and counties of Hainan, China.

**Results:**

All 231 centenarians had a mean age of 103.03 years. Survival participants had a longer CC and were often living alone compared with others (*P*<0.05 for all). Multivariate Cox regression models showed that CC was negatively associated with all-cause mortality (*P* < 0.05 for all). Participants with a longer CC had a lower mortality risk compared with others [Exp(β): 0.918; 95%confidence interval: 0.863–0.977]. Participants with a longer CC had a lower mortality risk whether they were males or females and lived with family members or alone.

**Conclusion:**

CC was negatively associated with all-cause mortality and could be an indicator of future mortality among the Chinese centenarians. Further researches should focus on preventing a decline in the CC in order to promote human longevity.

## Introduction

Sarcopenia is an aging-related disorder characterized by a loss of muscle mass and function. It might be associated with a wide range of illness, frailty, disability and mortality [1, 2]. Calf circumference (CC) is a surrogate marker of muscle mass and function [3]. CC is confirmed to be associated with conventional components of sarcopenia, including low muscle mass and function, and overlap percentage with other components is the greatest at low CC [4, 5]. As a commonly used indicator of anthropometric measurement, body mass index (BMI) is often used as a significant reference for nutrition and frailty [6], but in the recent years, studies have found that CC is more effective compared with BMI in assessing diseases risk in the older adults [7, 8]. Insufficient muscle mass and body fat redistribution, typically during the development of aging, could occur without changed BMI [9].

In the past century, median length of human life rose dramatically. According to a report, about 1 in 5,000 persons in United States are centenarians, and this prevalence is predicted to increase in the developed countries [10–12]. Even some analyses shows that the probability of females living more than 100 years in the future will reach 1 in 2. Interestingly, although the base of centenarians is expanding, the maximum life span of centenarians has not increased significantly. Identifying and intervening reversible risk factors for centenarian death has become an important way to break age limit of human [13, 14]. Growing evidence suggests that CC might be a simple but valuable screening tool in assessing mortality risk [15–17]. However, few studies have assessed the association between CC and mortality risk in the Asian population, especially in the centenarians. In this prospective study, we hypothesized that CC had significant association with the length of remaining life in the Chinese centenarians. To evaluate this hypothesis, we investigated the association between CC and all-cause mortality in the Chinese centenarians from a large population-based China Hainan Centenarian Cohort Study (CHCCS) during a follow-up for 5 years.

## Methods

### Study participants

**Fig. 1:**
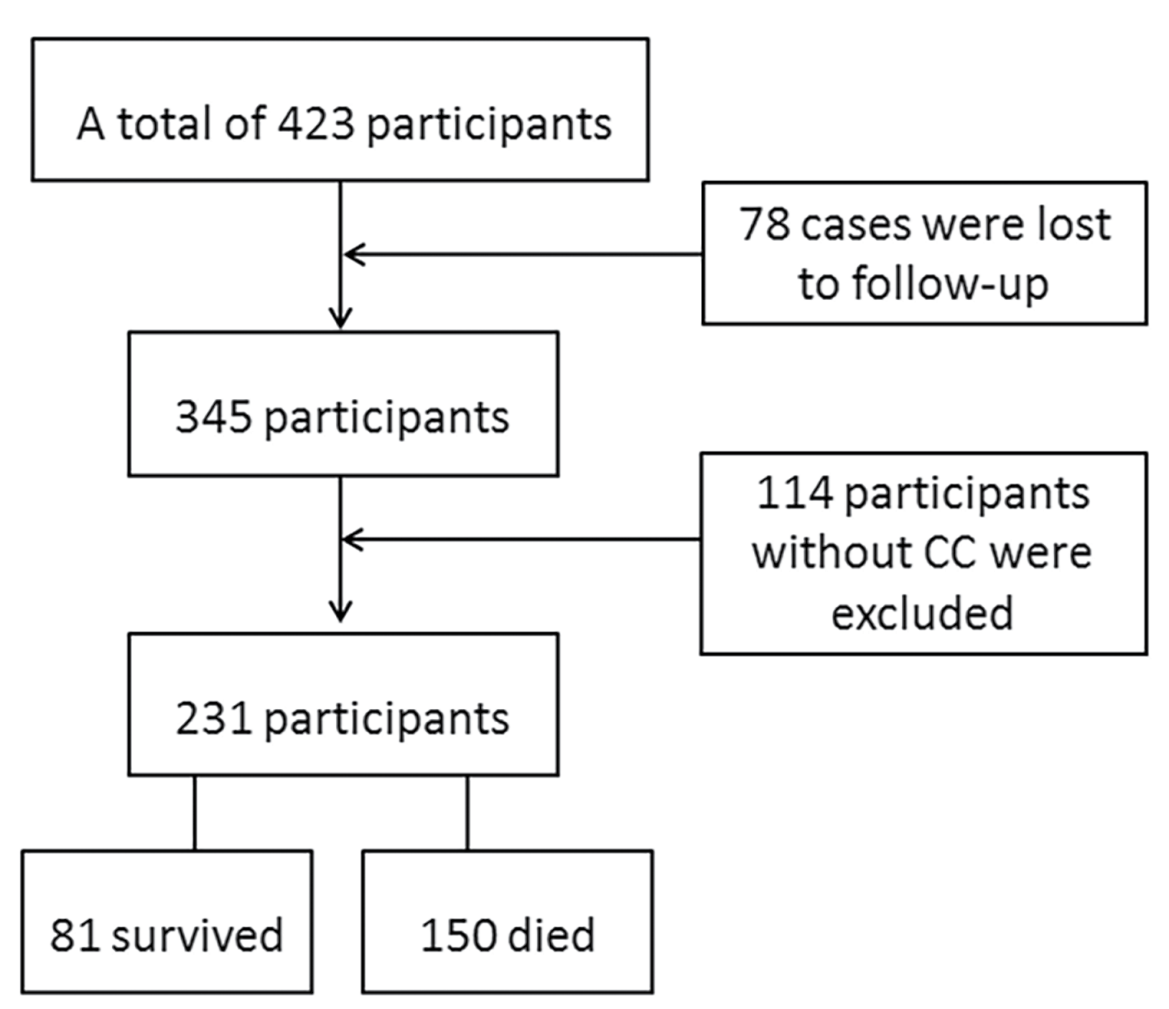
Flow chart of study

Based on the permission and support of Hainan Civil Affairs Bureau, the CHCCS is a prospective study conducted in 18 cities and counties of Hainan, China, from 2014 to 2016. Age is provided and verified by Hainan Civil Affairs Bureau, and the research design and survey methodology have been described previously [18]. A total of 423 centenarians were followed up, with 78 cases lost to follow-up. In all, 231 cases (81 survival centenarians and 150 dead centenarians) were included in this study, with 114 cases excluded due to data loss (Fig. 1). This study has been approved by the Ethics Committee of Hainan Hospital of Chinese People’s Liberation Army General Hospital (301HNLL-2016-01). The informed consents were obtained from all the participants and their legal guardians.

### Characteristic variables

Baseline data used in this study were obtained by questionnaire survey and physical examination following standard procedures [19, 20]. Questionnaire survey was conducted by face-to-face household interviews in a standardized format, with supplementary question-and-answers by family members of participants, by a strictly trained medical and nursing team familiar with local dialect [21, 22]. Questionnaire survey included more than 900 items. Age, sex, and ethnicity were subject to participant’s identity document card. Illiteracy was defined as a lack of formal cultural education. Residence was divided into living with family members or alone.

Physical examination was conducted by multidisciplinary physicians including geriatricians, neurologists, cardiologists, endocrinologists, nephrologists and nurses. A ruler was placed on the thickest part of the inspected centenarians’calf and abdomen, wrapping around the calf and abdomen for measurements in the horizontal direction and a standing position. Measurements were conducted by two operators and averaged, and all the results were counted in the centimeters. The operator measured systolic and diastolic blood pressure (SBP and DBP) of the inspected centenarians who took a sitting position by a calibrated desktop sphygmomanometer (Yuwell medical equipment and supply Co., Ltd., Jiangsu, China). Each parameter was measured twice and averaged, with a one-minute interval between two measurements. As the hospital specialist diagnoses determined by professional physicians who participated in this study, hypertension was defined by SBP ≥ 140 mmHg, DBP ≥ 90 mmHg or taking anti-hypertensive drugs, diabetes was defined by fasting blood glucose ≥ 7.0 mmol/L or taking hypoglycemic drugs/insulin, and cardiovascular disease (CVD) was defined by medical histories, clinical symptoms, cardiac markers and specific tests (e.g. electrocardiogram, echocardiogram, computed tomography and coronary arteriography) according to the American College of Cardiology/American Heart Association/European Society of Cardiology guidelines. Based on follow-up information obtained from telephone contact with these centenarians and their relatives, there were 150 (64.9%) deaths identified during a follow-up of almost 5 years. Follow-up time for the dead was defined from baseline to death date, and its time for the survival was defined at the end of follow-up.

### Statistical analyses

Statistic Package for Social Science version 26.0 (Chicago, IL, USA) was used for data management and statistical analyses. Continuous variable with normal distribution was represented using mean and standard deviation and compared using Student’s T test. Continuous variable with skewed distribution was represented using median and interquartile range and compared using Mann-Whitney U test. Categorical variable was represented by number and percentage and compared using Chi-Square test. Multivariate Cox regression models were conducted with independent variable of CC as a continuous variable and dependent variable of all-cause mortality in the following three models. Model 1 was unadjusted; model 2 was adjusted for common demographic factors including age, sex, and ethnicity; and model 3 was adjusted for potential risk factors including age, sex, ethnicity, illiteracy, residence, smoking, drinking, abdominal circumference, hypertension, diabetes, and CVD. Covariates were chosen in these models based on relevant authoritative literature and clinical experience. Stratified analyses were conducted by sex, education, residence, smoking, drinking, hypertension, diabetes, and CVD, and the significance of interaction was tested by a two-way interaction term in the final model. Results were represented as Exp(β) and 95% confidence intervals. A P value less than 0.05 was regarded as significant all through the analyses.

**Table 1:** Basic characteristics of included and excluded centenarians

Characteristics	Included (*n* = 231)	Excluded (*n* = 192)	*P*
Age, years	103.0 ± 2.9	102.8 ± 2.6	0.317
Sex			0.090
Males	41(17.7)	47(24.5)	
Females	190(82.3)	145(75.5)	
Ethnicity			0.725
Han	207(89.6)	170(88.5)	
Minority	24(10.4)	22(11.5)	
Education			0.462
None	215(93.1)	175(91.1)	
1 year or more	16(6.9)	17(8.9)	
Residence			0.948
With family members	198(85.7)	165(85.9)	
Alone	33(14.3)	27(14.1)	
Smoking			0.343
Yes	19(8.2)	21(10.9)	
No	212(91.8)	171(89.1)	
Drinking			0.264
Yes	31(13.4)	19(9.9)	
No	200(86.6)	173(90.1)	
Hypertension			0.845
Yes	168(72.7)	138(71.9)	
No	63(27.3)	54(28.1)	
Diabetes			0.970
Yes	25(10.8)	21(10.9)	
No	206(89.2)	171(89.1)	
Cardiovascular disease			0.340
Yes	10(4.3)	5(2.6)	
No	221(95.7)	187(97.4)	

## Results

### Basic characteristics of total participants

Total participants had a mean (standard deviation) age of 103.03 (2.93) years. Females accounted for 82.25% (190 participants). Table 1 showed that basic characteristics had no difference between included and excluded centenarians (*P* > 0.05 for all). As shown in Table 2, survival participants had a longer CC and were often living alone compared with others (*P*<0.05 for all).

**Table 2:** Basic characteristics of survival and dead centenarians

Characteristics	All-cause mortality		*P*
	Survival (*n* = 81)	Dead (*n* = 150)	
Calf circumference, cm	25.5 ± 3.0	24.6 ± 3.2	0.034
Age, years			0.254
100–105	64(79.0)	124(82.7)	
106–110	12(14.8)	23(15.3)	
111–115	5(6.2)	3(2.0)	
Sex			0.223
Males	11(13.6)	30(20.0)	
Females	70(86.4)	120(80.0)	
Ethnicity			0.474
Han	71(87.7)	136(90.7)	
Minority	10(12.4)	14(9.3)	
Education			0.156
None	78(96.3)	137(91.3)	
1 year or more	3(3.7)	13(8.7)	
Residence			0.011
With family members	63(77.8)	135(90.0)	
Alone	18(22.2)	15(10.0)	
Smoking			0.404
Yes	5(6.2)	14(9.3)	
No	76(93.8)	136(90.7)	
Drinking			0.389
Yes	13(16.1)	18(12.0)	
No	68(84.0)	132(88.0)	
Abdominal circumference, cm	76.6 ± 8.2	75.5 ± 9.2	0.367
Hypertension			0.778
Yes	58(71.6)	110(73.3)	
No	23(28.4)	40(26.7)	
Diabetes			0.917
Yes	9(11.1)	16(10.7)	
No	72(88.9)	134(89.3)	
Cardiovascular disease			0.731
Yes	3(3.7)	7(4.7)	
No	78(96.3)	143(95.3)	

### Association between CC and all-cause mortality

Table 3 showed that CC was negatively associated with all-cause mortality after gradually adjusting the covariates in the multivariate Cox regression models (*P* < 0.05 for all). Participants with a longer CC had a lower mortality risk compared with others [Exp(β): 0.918; 95%confidence interval: 0.863–0.977].

**Table 3:** Association between calf circumference and all-cause mortality in the multivariate Cox regression models

Variables	β	Exp(β)	95%confidence interval	*P*
Model 1	-0.065	0.937	0.887–0.989	0.019
Model 2	-0.093	0.911	0.861–0.964	0.001
Model 3	-0.085	0.918	0.863–0.977	0.007

Model 1: independent variable was calf circumference as a continuous variable and dependent variable was all-cause mortality without adjustment;

Model 2: with the adjustment of age, sex, and ethnicity;

Model 3: with the adjustment of age, sex, ethnicity, illiteracy, residence, smoking, drinking, abdominal circumference, hypertension, diabetes, and cardiovascular disease

### Stratified interaction of CC and mortality

**Fig. 2:**
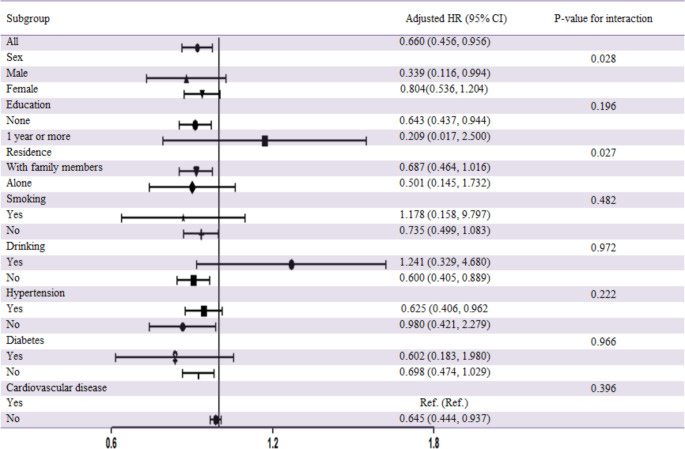
Association between calf circumference and all-cause mortality stratified by participant characteristics

Figure 2 showed significant between-group differences in the association between CC and all-cause mortality stratified by sex and residence in the multivariate Cox regression models (*P*<0.05 in interaction for all). Participants with a longer CC had a lower mortality risk compared with others whether they were males or females and lived with family members or alone.

## Discussion

In this study, we demonstrated that CC was negatively associated with all-cause mortality in a representative cohort of Chinese centenarians. After adjusting the covariates, this association remained unchanged. In addition, stratified analyses suggested that sex and residence did not affect the association between CC and all-cause mortality in these Chinese centenarians.

Our findings suggested that to achieve an increase in the CC could be an effective tool for promoting human longevity. These results agreed with previous reports on the association between CC and mortality in the older adults. A cohort study including older adults more than 65 years showed that the highest quartile of CC was associated with a lower mortality risk compared with the second quartile, while the lowest quartile had the opposite result [8, 23, 24]. A retrospective cohort study included participants with a mean (standard deviation) age of 50.6 (18.5) years showed that increased CC was associated with a lower mortality risk [25]. A meta-analysis found that reduced CC was associated with a higher mortality risk compared with others, and the association remained unchanged after adjusting for different settings (hospital and community) [16]. In particular, Shou’s study found that a shorter CC increased sarcopenic components correlating with all-cause mortality [26]. Mortality risk associated with CC might be related to a decrease in the insulin sensitivity index and an increase in the visceral fat thickness [27, 28].

CC as an evaluation indicator for muscle mass and function has been found to be closely related to several diseases in the recent years [29, 30]. Previous study found that a shorter CC was associated with increased mortality risk [31]. In short, changed body homeostasis caused by a decline in the CC might also be a factor leading to increased mortality risk [32, 33]. One novelty of this study is that our study participants consisted of centenarians. While highlighting the findings, there had several limitations. First, this study is based on a cohort study of centenarians in Hainan, China, and all the participants are Chinese, so these results might not apply to others. Second, CC could not be obtained completely since many centenarians were frail. Third, whether this study could be extended to younger adults still needs to be confirmed with further study.

## Conclusion

This prospective study demonstrated among the Chinese centenarians that CC was negatively associated with all-cause mortality and could be an indicator of future mortality. Further researches should focus on preventing a decline in the CC in order to promote human longevity.

## Data Availability

The authors confirm that data supporting the findings of this study are available within the article.
